# Electron Irradiation of Metal Contacts in Monolayer MoS_2_ Field-Effect Transistors

**DOI:** 10.1021/acsami.0c11933

**Published:** 2020-08-10

**Authors:** Aniello Pelella, Osamah Kharsah, Alessandro Grillo, Francesca Urban, Maurizio Passacantando, Filippo Giubileo, Laura Iemmo, Stephan Sleziona, Erik Pollmann, Lukas Madauß, Marika Schleberger, Antonio Di Bartolomeo

**Affiliations:** † Department of Physics and Interdepartmental Centre NanoMates, University of Salerno, via Giovanni Paolo II, Fisciano 84084, Italy; ‡ CNR-SPIN, via Giovanni Paolo II, Fisciano 84084, Italy; § INFN—Gruppo Collegato di Salerno, via Giovanni Paolo II, Fisciano 84084, Italy; ∥ Department of Physical and Chemical Sciences, University of L’Aquila, and CNR-SPIN L’Aquila, via Vetoio, Coppito, L’Aquila 67100, Italy; ⊥ Fakultät für Physik and CENIDE, Universität Duisburg-Essen, Lotharstrasse 1, Duisburg 47057, Germany

**Keywords:** molybdenum disulfide, field-effect transistors, Schottky barrier, scanning electron microscopy, Raman spectroscopy, photoluminescence, electron beam irradiation, electron interactions in solids

## Abstract

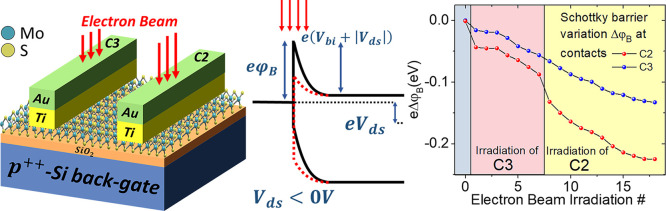

Metal contacts play a fundamental role in nanoscale devices. In this work, Schottky metal contacts in monolayer molybdenum disulfide (MoS_2_) field-effect transistors are investigated under electron beam irradiation. It is shown that the exposure of Ti/Au source/drain electrodes to an electron beam reduces the contact resistance and improves the transistor performance. The electron beam conditioning of contacts is permanent, while the irradiation of the channel can produce transient effects. It is demonstrated that irradiation lowers the Schottky barrier at the contacts because of thermally induced atom diffusion and interfacial reactions. The simulation of electron paths in the device reveals that most of the beam energy is absorbed in the metal contacts. The study demonstrates that electron beam irradiation can be effectively used for contact improvement through local annealing.

## Introduction

Molybdenum disulfide (MoS_2_) is one of the most studied transition metal dichalcogenides, owing to its layered structure and useful mechanical, chemical, electronic, and optoelectronic properties.^1−4^ A molybdenum (Mo) atomic plane sandwiched between two sulfur (S) planes constitutes the monolayer that is bonded to other monolayers by weak van der Waals forces to form the bulk material. MoS_2_ is a semiconductor suitable for several applications,^5−9^ having a 1.2 eV indirect band gap in the bulk form that widens up to 1.8–1.9 eV and becomes direct in the monolayer.^3^ Despite the lower field-effect mobility than graphene,^10,11^ ranging from few tenths to hundreds^12−15^ of cm^2^ V^–1^ s^–1^, MoS_2_ field-effect transistors (FETs) have recently become very popular as alternatives to graphene FETs^12−17^ for next-generation electronics based on 2D materials.^18−25^

The fabrication and characterization of devices based on 2D materials greatly rely on the application of electron beam (e-beam) lithography or focused ion beam processing and on scanning electron microscopy (SEM) or transmission electron microscopy, which imply irradiation by charged particles. The exposure to low-energy electrons and/or ions can modify the electronic properties of the 2D materials or their interfaces.^9,17,26^ Indeed, structural defects can locally modify the band structure and behave as charge traps, thereby changing the device characteristics both in the case of e-beam^27,28^ and ion beam irradiation.^29,30^ Conversely, electron beam, ion irradiation, or plasma treatments can be intentionally used for nanoincisions,^31^ for pores,^32^ or to purposely create defects, for instance, to reduce the contact resistance.^33−35^ Choi et al. reported the effects of 30 keV electron beam irradiation of monolayer MoS_2_ FETs, showing that irradiation-induced defects act as trap sites by reducing the carrier mobility and concentration and shifting the threshold voltage.^36^ A study of point defects in MoS_2_ using SEM imaging and first-principles calculations, by Zhou et al., demonstrated that vacancies are created by e-beam irradiation at low energies,^37^ below 30 keV. Durand et al. studied the effects of e-beam on the MoS_2_-based FET, reporting an increase in carrier density and a decrease in mobility explained as irradiation-induced generation of intrinsic defects in MoS_2_ and as Coulomb scattering by charges at the MoS_2_–SiO_2_ interface, respectively.^38^ Giubileo et al. reported a negative threshold voltage shift and a carrier mobility enhancement under 10 keV electron irradiation of few-layer MoS_2_ FETs attributed to beam-induced positive charge trapped in the SiO_2_ gate oxide.^27^

In this paper, we present the spectroscopic and electrical characterization of monolayer MoS_2_-based FETs, with Schottky Ti/Au contacts, focusing on the effects of low-energy e-beam irradiation. We show that the long exposure of the metal contacts to 10 keV e-beam in a SEM chamber enhances the transistor’s on-current. We explain such an improvement by radiation-induced lowering of the Schottky barrier at the metal contacts. We perform Monte Carlo simulation to track the e-beam through the device and show that when the beam is focused onto the contacts, most of the beam energy is absorbed within the metal. The local heat can induce atomic diffusion and interfacial reactions that change the chemical composition and structure of the metal–MoS_2_ interface or can generate or release tensile strain. Both effects cause the lowering of the Schottky barrier and the consequent increase in transistor current.

Our study shows that electron beam exposure during SEM imaging has non-negligible effects on MoS_2_ devices; however, it also highlights that a suitable exposure, with the e-beam focused on the contact region, can be conveniently exploited to reduce the contact resistance of the transistor. Compared to thermal annealing, our finding provides a way to improve the contact resistance by local conditioning, which avoids the exposure of the entire wafer to a high thermal budget.

## Fabrication and Experimental Methods

The MoS_2_ monolayer flakes were grown via chemical vapor deposition in a three-zone split tube furnace, purged with 500 Ncm^3^/min Ar gas for 15 min to minimize the O_2_ content. The growth SiO_2_/Si substrate was spin-coated with a 1% sodium cholate solution; then, a saturated ammonium heptamolybdate (AHM) solution was first annealed at 300 °C under ambient conditions to turn AHM into MoO_3_ to be used as the source for molybdenum. The target material was placed in a three-zone tube furnace along with 50 mg of S powder, positioned upstream in a separate heating zone. The zones containing S and AHM were heated to 150 °C and 750 °C, respectively. After 15 min of growth, the process was stopped, and the sample was cooled rapidly.

**Figure 1:**
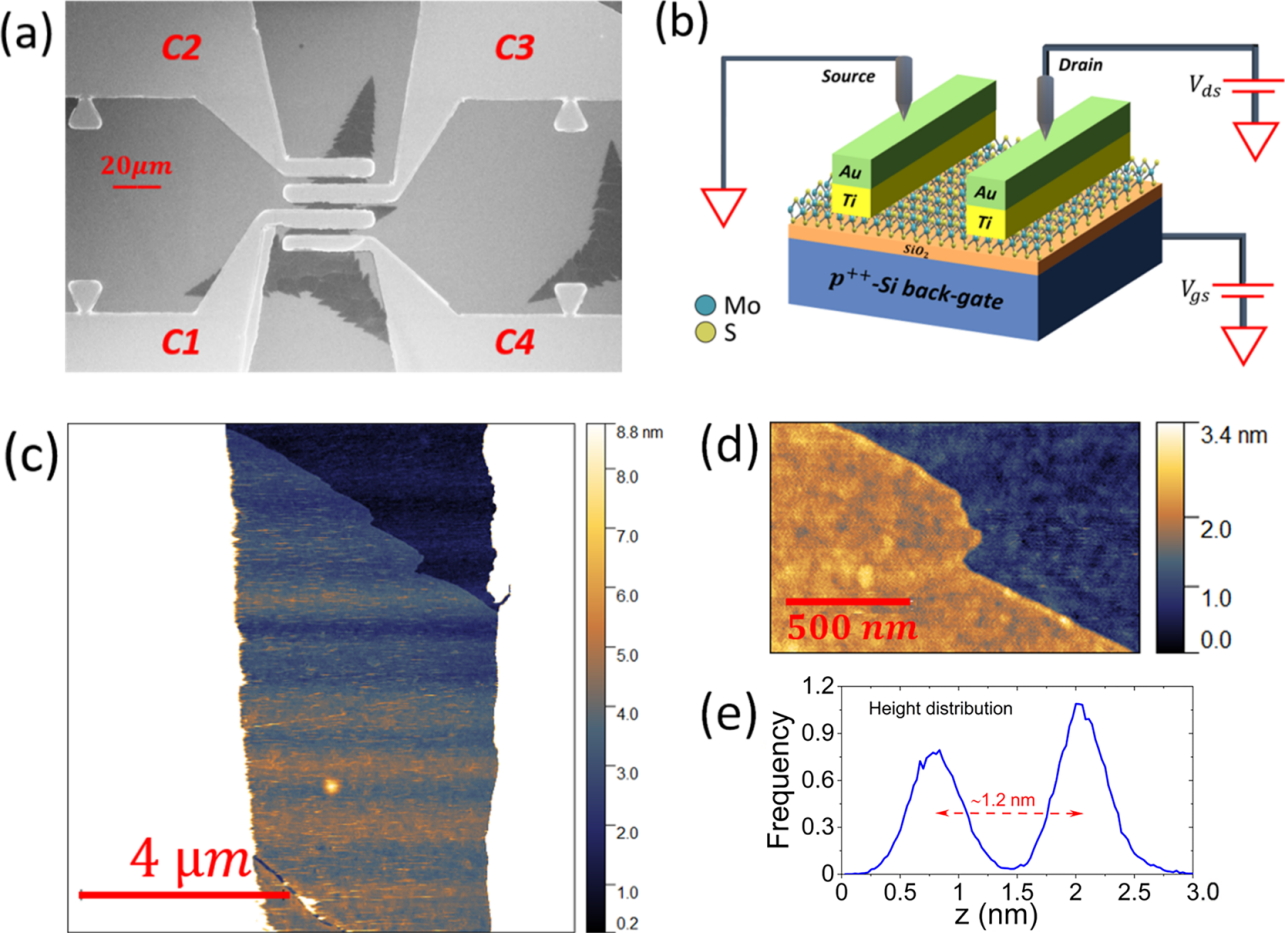
(a) SEM image of the MoS_2_ device and contact labels. (b) MoS_2_ FET layout and schematic of the common source configuration used for electrical characterization. (c) AFM image of the MoS_2_ flake between the electrical contacts, which appear here in white as the scale has been adjusted to properly image the MoS_2_ flake. (d) Zoom-in into the upper region of (c), showing that the flake is flat and structurally intact. The rms roughness is 0.221 nm for the SiO_2_ substrate and 0.237 nm for MoS_2_. (e) Height distribution taken from image (d), yielding a flake height of ∼1.2 nm.

We realized FETs using the SiO_2_/Si substrate (thickness of the dielectric: 285 nm) as the back gate and evaporating the drain and source electrodes on selected MoS_2_ flakes through standard photolithography and lift-off processes. The contacts were made of Ti (10 nm) and Au (40 nm) used as adhesion and cover layers, respectively. Ti was deposited in high vacuum, which could not exclude the formation of TiO_2_, contributing to the resistance and Schottky barrier at the contacts. Figure 1a,b shows the SEM top view of a typical device and its schematic layout and measurement setup. The channel is made up from a monolayer flake [as confirmed by Raman and photoluminescence (PL), see below] of width and length of 20 and 4 μm, respectively, and a nominal thickness of 0.7 nm. Atomic force microscope (AFM) images (Figure 1c,d,e) show that the flake has an average height of 1.2±0.3 nm (which is typical for single-layer MoS_2_ measured in air by AFM) and appears to be extremely flat (roughness rms < 0.25 nm) and structurally intact. There are some contaminants because of the lithography process, which are weakly bound and can be swept by the AFM tip. Contacted and noncontacted flake areas do not differ with respect to contamination density—spectroscopic data should thus be comparable.

**Figure 2:**
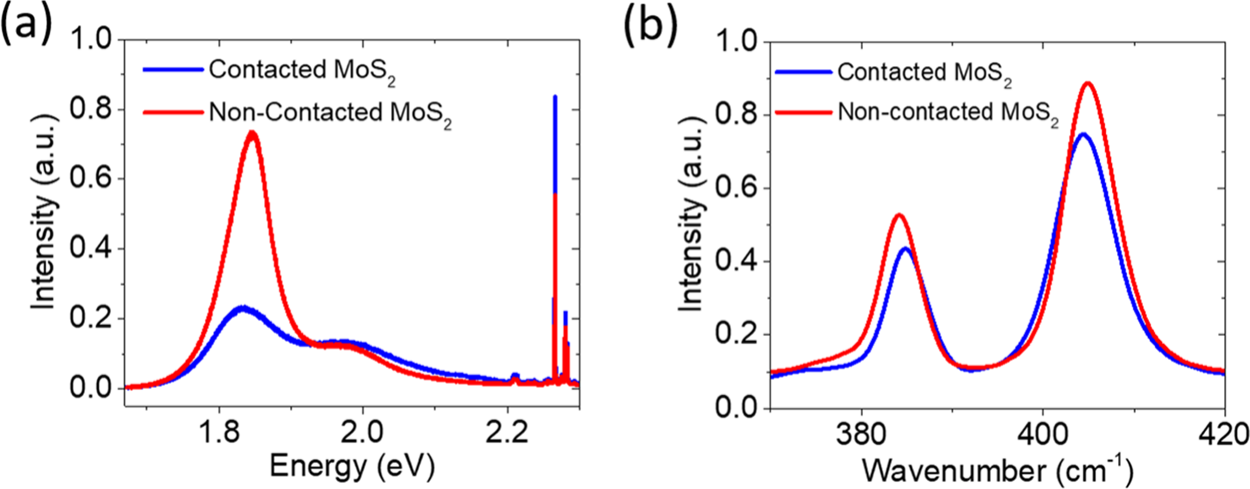
(a) PL and (b) Raman spectrum of monolayer MoS_2_ after FET processing. Blue: contacted MoS_2_ monolayer flake and red: noncontacted monolayer MoS_2_ flake.

A total of seven MoS_2_ channels of identically prepared FETs have been characterized by Raman and PL spectroscopy just after processing. The measurements were performed with a Renishaw InVia Raman microscope at the Interdisciplinary Center for Analytics on the Nanoscale (ICAN). The excitation laser wavelength was 532 nm, and the power density was kept below 0.1 mW/μm^2^ to avoid damage to the MoS_2_ flake. Exemplary spectra of Raman characterization are shown in Figure 2. The chosen reference measurements are spectra obtained from MoS_2_ flakes on the same substrate, which were also in contact with the photoresist and various solvents during the processing and lift-off for the production of the FETs, but are not in contact with metal electrodes themselves. The shape of the PL spectra (Figure 2a) and the difference of the Raman modes (Figure 2b) differ significantly. The PL intensity (sum of all excitons and trions) for noncontacted MoS_2_ flakes is higher by a factor of 1.7 ± 0.8 than that for contacted MoS_2_. The mode differences for noncontacted and contacted MoS_2_ are 21.3 ± 0.7 cm^–1^ and 19.7 ± 0.7 cm^–1^, respectively. Both the changes in PL and Raman mode difference can be associated with built-in strain or changes in the electronic properties and the band structure of the MoS_2_ sheets.^39−43^ From the linear dependencies of Raman mode positions on doping and strain,^39,40^ we find a reduction of tensile strain by (0.46 ± 0.28) % and an increase in electron doping of 0.44 ± 0.36 × 10^13^ electrons per cm^2^ for the contacted 2D material in comparison with noncontacted MoS_2_ (details of the calculation method can be found in ref (44)). Hence, the significant alterations in the spectroscopic pre-characterization of the MoS_2_ channels can be clearly attributed to electronic and structural changes at the metal contact.

In the following, most of the electrical characterization refers to the transistor between the contacts labeled C2 and C3 in Figure 1a. The contact C3 was used as the drain and C2 as the grounded source. The electrical measurements were carried out inside a SEM chamber (LEO 1530, Zeiss), endowed with two metallic probes with nanometer positioning capability, connected to a Keithley 4200 SCS (source measurement units, Tektronix Inc.), at room temperature and a pressure of about 10^–6^ mbar. The e-beam of SEM, set to 10 keV and 10 pA, was used for the time-controlled irradiation of specific parts of the device.

## Results and Discussion

**Figure 3:**
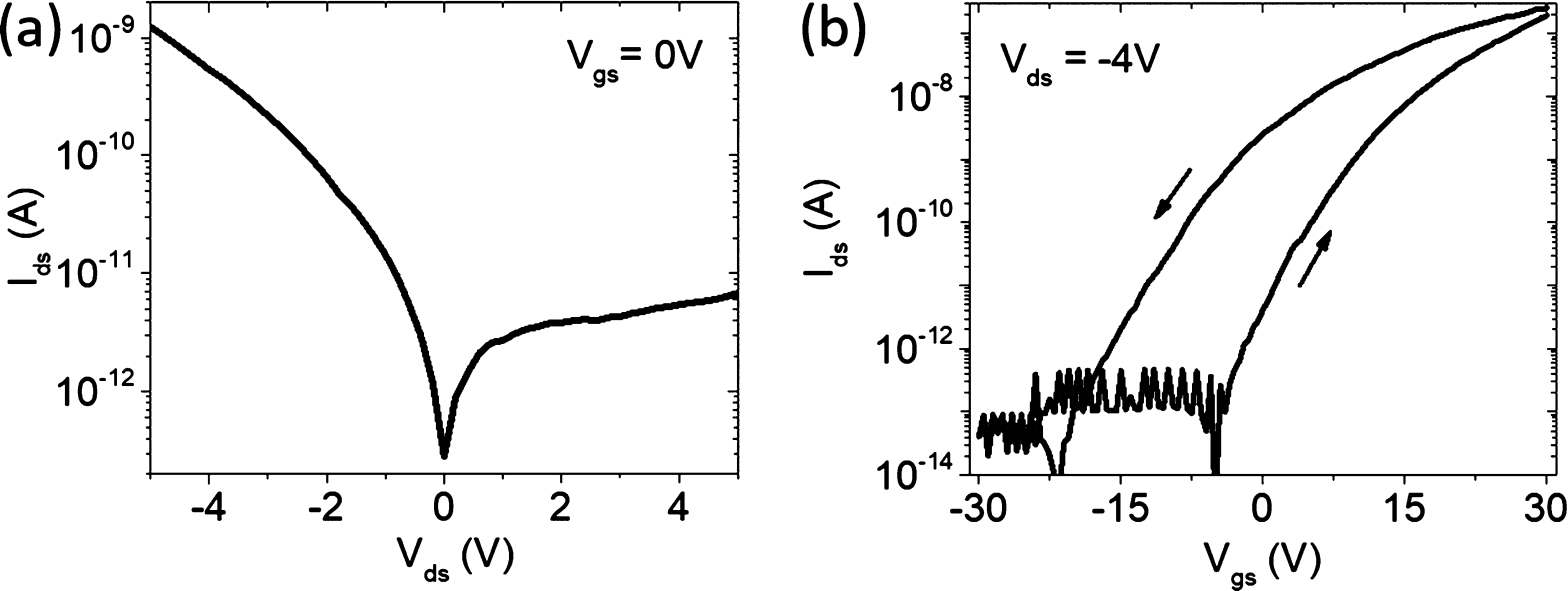
Output (a) and transfer (b) characteristics of the device between C2 and C3 contacts, with C3 used as the drain and C2 as the grounded source.

The output (*I*_ds_–*V*_ds_) and the transfer (*I*_ds_–*V*_gs_) characteristics of the transistor are shown in Figure 3a,b, respectively. The output curve shows rectification with the forward current appearing at negative *V*_ds_, typical of a p-type Schottky diode, while the transfer characteristic shows an n-type transistor. This apparently contradictory behavior has been previously reported for MoS_2_ and WSe_2_ transistors and explained by the formation of two back-to-back and possibly asymmetric Schottky barriers at the contacts.^45,46^ The forward current at negative *V*_ds_ is caused by the different contact areas and by the image force barrier lowering of the forced junction (i.e., the drain, C3, in our case), while the reverse current at *V*_ds_ > 0 V is limited by the grounded junction at the source (C2) contact. As the barrier lowering is more effective on the forced junction, the voltage being directly applied to it, the negative bias gives rise to the higher (apparently forward) current.

After the initial electrical characterization, we performed two sets of exposures to the SEM electron beam. Each exposure lasted 300 s, corresponding to a fluence of ∼180 e^–^/nm^2^, over a surface area of ∼100 μm^2^. The two sets of irradiations were carried out first on the drain contact (C3) and then on the grounded source contact (C2). A final exposure of the MoS_2_ channel to the e-beam was performed as well.

**Figure 4:**
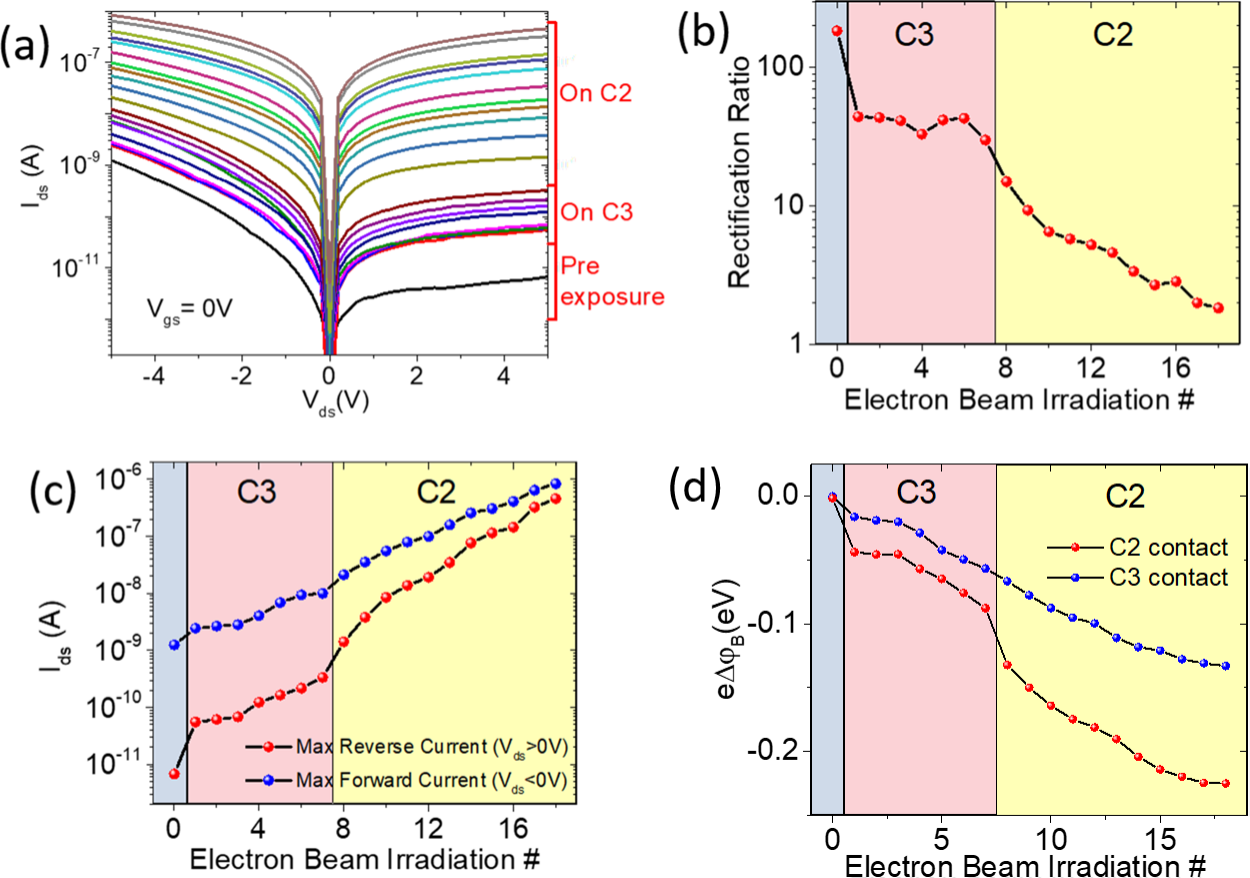
(a) Output characteristics at *V*_gs_ = 0 V of the transistor formed by contacts C2–C3 exposed to two sets of electron irradiations performed first on contact C3 and then on C2. (b) Rectification ratio and (c) maximum forward and reverse current, at *V*_ds_ = ±5 V, as a function of the irradiation number. (d) Zero-bias Schottky barrier variation at the contacts C2 and C3 as a function of the irradiation number.

Figure 4 summarizes the obtained results. The *I*_ds_–*V*_ds_ curves were measured at the end of each irradiation, ∼120 s after the blanking of the e-beam, to allow cooling down. Starting from the bottom (black) line in Figure 4a, representing the output curve of the unexposed device, the current increases with the e-beam exposures. We note two major discontinuities in the sequence of *I*_ds_–*V*_ds_ curves, corresponding to the start of the two irradiations sets. These gaps are likely due to the uncontrolled exposure of the whole device during the selection of the drain (C3) and grounded source (C2) contact areas for the respective irradiation sets.

A different behavior of the forward with respect to the reverse current can be observed in Figure 4a, and a distinction of the effects of the irradiations on the drain (C3) and the grounded source (C2) can be made. Although the irradiation of the drain increases both the forward and the reverse currents, keeping the rectification ratio almost constant (see Figure 4 b), the irradiation of the source augments the reverse current in a faster way, rendering the output curves more symmetric. Figure 4b shows that repeated irradiations of the drain contact (C3) do not change the rectification ratio (at *V*_ds_ = ±5 V), while the irradiation of the grounded source contact (C2) dramatically decreases the rectification ratio. Figure 4c shows that the maximum reverse and forward currents, at *V*_ds_ = ±5 V, have different variation rates when the irradiation is either on the drain or source. Noticeably, Figure 4c shows that the increase in both the reverse and forward currents is an exponential function of the fluence, which is proportional to and can be parametrized by the irradiation number.

As the shape and the current intensity of the output characteristics are related to the Schottky barrier heights at the contacts, the exponentially increasing current and the changing rectification ratio point to radiation-induced Schottky barrier lowering. The energy release in the metal contacts can modify the chemistry of the metal–MoS_2_ interface or create stress and defects that can lead to a lowering of the barrier and a consequent contact resistance reduction. We note that the reduction of contact resistance by chemical reactions between the metal contacts and MoS_2_ channel has been reported for the metal deposited under ultrahigh vacuum^47^ and contact laser annealing.^48^ A disordered, compositionally graded layer, composed of Mo and Ti_*x*_S_*y*_ species, forms on the surface of the MoS_2_ crystal following the deposition of Ti, and thermal annealing in the 100–600 °C temperature range can cause Ti diffusion inducing further chemical and structural changes at the Ti–MoS_2_ interface.^49,50^ It is also possible that diffusion of Au atoms to the interface with MoS_2_ occurs under the energetic electron beam irradiation. Au does not react with MoS_2_ but reduces the contact resistance and therefore the Schottky barrier height.^51^

Similarly, tensile strain has been demonstrated to induce considerable Schottky and tunneling barrier lowering.^52^

A Schottky barrier of ∼0.2 eV is formed by several metals on MoS_2_ because of Fermi level pinning below the MoS_2_ conduction band.^53−55^ Density functional theory calculations have indicated that the pinning at the metal–MoS_2_ interface is different from the well-known Bardeen pinning effect, metal-induced gap states, and defect/disorder-induced gap states, which are applicable to traditional metal–semiconductor junctions. At metal–MoS_2_ interfaces, the Fermi level is pinned either by a metal work function modification due to interface dipole formation arising from the charge redistribution or by the production of gap states mainly of Mo d-orbitals, characterized by the weakened intralayer S–Mo bonding because of the interface metal–S interaction.^56,57^ The observed decrease in the Schottky barrier by e-beam irradiation, up to its complete disappearance, supports the occurrence of interface modifications that cause Fermi level depinning.

As the forward current at *V*_ds_ < 0 V is limited by the Schottky barrier at the drain contact (C3), while the reverse current at *V*_ds_ > 0 V is limited by the Schottky barrier at the grounded source contact C2 (which are the reverse-biased junctions for negative and positive *V*_ds_, respectively), the output curves of Figure 4a, which correspond always to reverse current, can be used to extract the behavior of the Schottky barriers as a function of the fluence (i.e., the e-beam irradiation number). Let us consider the thermionic current through a reverse-biased Schottky barrier^58,59^
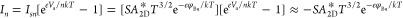
1where φ_B*n*_ and *I*_s*n*_ are the barrier height and the reverse saturation current at the n-th e-beam irradiation, *S* is the junction area, *A*_2D_^*^ is the 2D Richardson constant, *k* is the Boltzmann constant, *T* is the temperature, *n* is the ideality factor, and *V*_a_ is the negative voltage across the barrier that makes . Let us define *I_0_* as the reverse saturation current before e-beam exposure, that is, associated to the maximum barrier height φ_B0_. To avoid the effect of bias which can induce image-force barrier lowering,^60^ both *I*_*n*_ and *I*_0_ are obtained by extrapolating the measured currents to zero bias. Then, eq 1 can be used to evaluate the variation of the Schottky barrier, Δφ_B*n*_ = φ_B*n*_ – φ_B0_, as a function of the irradiation number
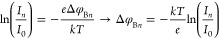
2

The zero-bias Schottky barrier variation, Δφ_B*n*_, is shown in Figure 4d for both source (C2) and drain (C3) contacts. The overall reduction of both barriers is comparable to the expected initial barrier height based on Fermi level pinning, meaning that the long irradiation can completely remove the barriers. The plot indicates that the two barriers behave differently for the irradiation of C2 or C3. Although the beam irradiation of either contact results in a lowering of both Schottky barriers, the barrier decrease is faster for the irradiation of the grounded source. Besides, the Schottky barrier at the source contact is the most affected by the irradiation of the source.

**Figure 5:**
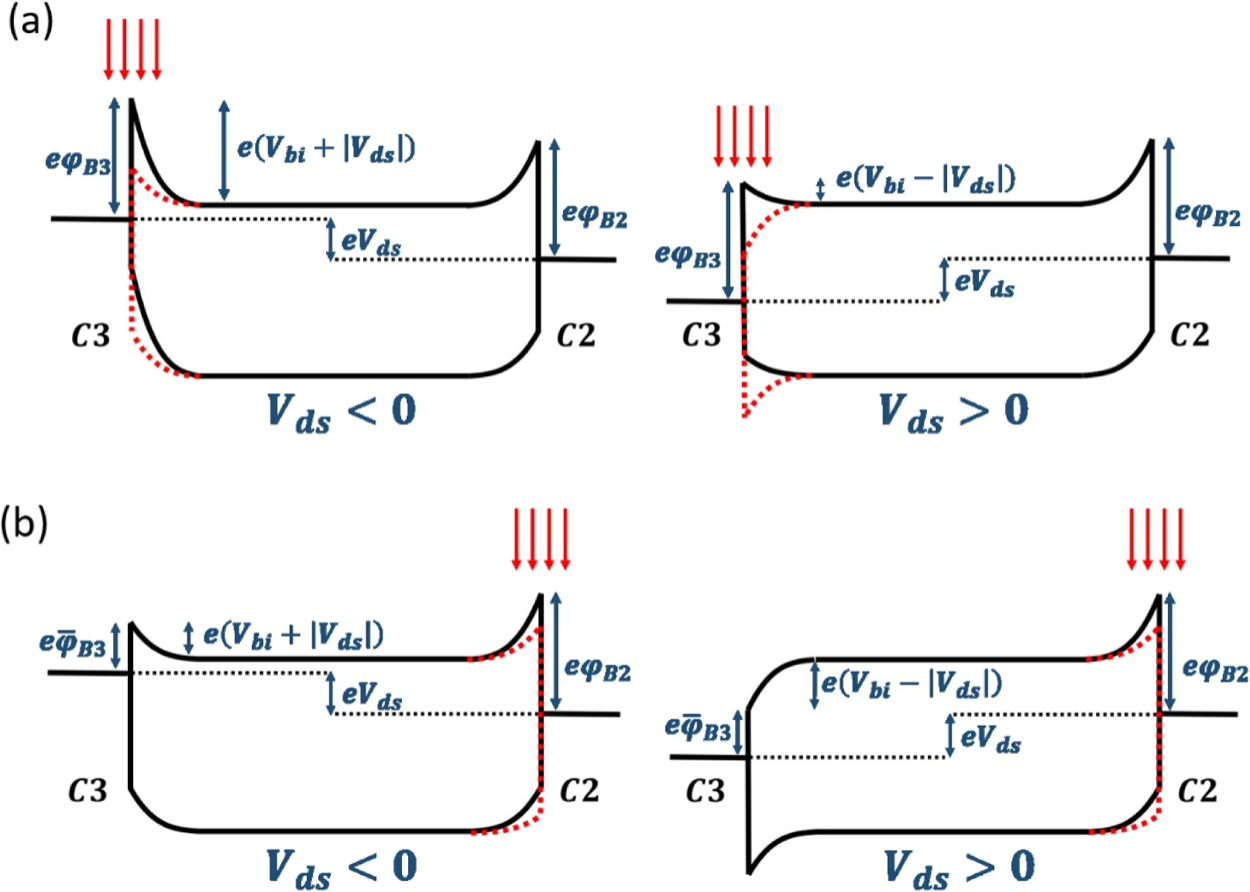
Low-bias energy band diagrams (black) and their modification under electron irradiation (red) of C3 (a) and of C2 (b) contacts resulting in barrier lowering (φ̅_B_).

To explain these results, we propose the model based on the energy band diagrams, shown in Figure 5. A negative (positive) voltage applied to the drain contact (C3) causes an upward (downward) shift of the energy bands in the drain region. Electron beam irradiation of the contact lowers the Schottky barrier and the relative built-in potential, as shown by the red dashed lines in Figure 5. The reduction of a Schottky barrier and of its associated built-in potential, at the irradiated contact, results also in the lowering of the unexposed barrier, which can experience a stronger potential drop because of the reduced contact resistance of the first contact. Figure 5a represents the situation in which the e-beam is focused on the biased drain contact (C3). At *V*_ds_ < 0 V, the current is limited mainly by the drain contact barrier which is lowered by the successive irradiations, causing the exponential increase in maximum forward current. At *V*_ds_ > 0 V, the current is limited by the un-irradiated source contact (C2) barrier, and its dependence on the irradiation cycle is caused by the lowering of the built-in potential at the drain (C3). As the barrier and built-in lowering are the same, the rectification ratio remains almost constant. For irradiation of the grounded source (C2, Figure 5b), the current increases because of a similar mechanism, with the difference that the drain contact barrier limits the current for *V*_ds_ > 0 V to a lesser extent, having been already irradiation-lowered. Therefore, the reverse current increases faster with the repeated irradiation and the rectification ratio decreases.

**Figure 6:**
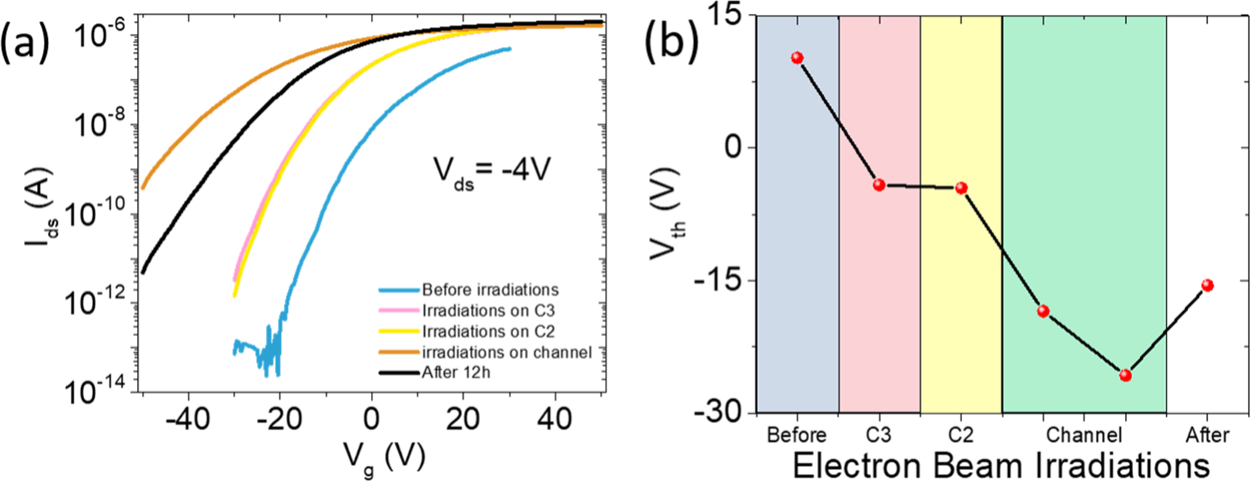
(a) FET transfer characteristics at *V*_ds_ = −4 V before and after e-beam irradiations of contacts C3 and C2 and of the channel. (b) Left shift of the threshold voltage extrapolated from the transfer characteristics over the e-beam exposure.

The effect of irradiation on the transfer characteristic of the transistor is shown in Figure 6 and confirms the radiation-induced increase in channel current. Besides, Figure 6a shows that the e-beam, independent of onto which contact it is focused on, causes a left shift of the transfer curve. Such a shift corresponds to a decrease in threshold voltage, defined as the x-axis intercept of the linear fit of the transfer curve on the linear scale. The threshold voltage as a function of the irradiation is displayed in Figure 6b. Although the e-beam exposure of the contacts provokes a left shift (the transfer curves are taken at the end of the two irradiation sets on the drain (C3) and grounded source (C2)), further left shift of the threshold voltage is observed when two successive irradiations are performed in the channel region.

The observed negative shift of the threshold voltage has been reported and discussed before.^27^ It can be explained by the pile-up of positive charge in trap states of the SiO_2_ gate dielectric or at the SiO_2_–Si interface. The e-beam exposures produce electron–hole pairs in the SiO_2_ gate oxide and in the Si substrate: although mobile electrons are easily swept by the applied bias, the positive charges can be stored for long times.^27^ The positive charge storage acts as an extra gate (similarly to the gating effect under light irradiation^61,62^) and enhances the n-type doping of the channel.

Indeed, Figure 6 shows that there is a slight recovery of the threshold voltage after 12 h of annealing at room temperature. However, we highlight that, as demonstrated by Figure 6a, the maximum channel current, which is limited by the contact resistances, remains unchanged after annealing, demonstrating that the irradiation-induced improvement of the contacts is permanent.

**Figure 7:**
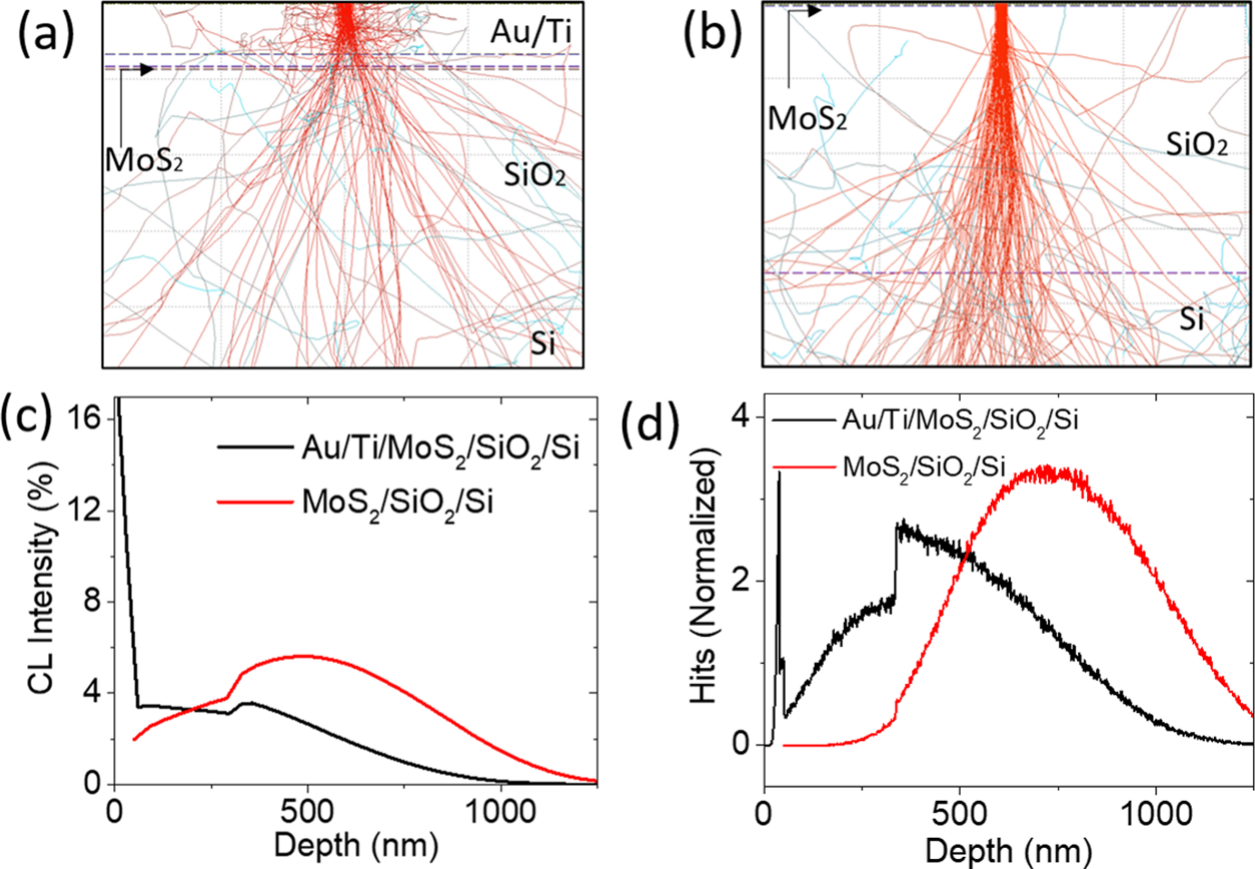
Monte Carlo simulation using CASINO v2 of e-beam irradiation of the device (a) contacts and (b) of the MoS_2_ channel. (c) Simulated cathodoluminescence intensity through the sample, with the e-beam focused onto the contacts and the flake. (d) Simulation of the electrons' penetration depth through the sample.

To further confirm our model, we performed a Monte Carlo simulation to track the path of the electrons under the contacts and in the channel region (Figure 7a,b), using the CASINO software package.^63−65^ We simulated a 10 keV beam with one million electrons and a radius beam of 10 nm. The cathodoluminescence spectrum (Figure 7c) shows that electrons lose their energy and are stopped (Figure 7d) mostly in the Ti/Au metal stack, while they reach and are absorbed in the Si substrate when the irradiation is on the channel. The high release of energy in the metal contacts, similarly to thermal annealing,^66,67^ induces Ti–MoS_2_ reactions and creates contact with the reduced Schottky barrier and contact resistance. Conversely, when we directly irradiate the MoS_2_ channel, energy is prevalently adsorbed in the Si bulk and its effect manifests only through the positive charge traps generated in the SiO_2_ layer.

## Conclusions

We investigated the effects of 10 keV electron beam irradiation of the Schottky metal contacts in MoS_2_-based FETs. Spectroscopic analysis by Raman and PL shows that the presence of metal contacts changes the properties of monolayer MoS_2_ with respect to strain and doping. The electrical measurements revealed that electron beam irradiation improves the device conductance, reduces the rectification of the output characteristic, and causes a left shift of the threshold voltage. To explain such a feature, we propose that the energy absorbed in the metal contacts induces atomic diffusion and interfacial reactions that lower the Schottky barrier at the contacts and improve the contact resistance. We corroborate our model by direct measurement of the Schottky barrier height variation and by simulation of the electron trajectories in the contact regions.
